# Ethylene Induced by Sound Stimulation Enhances Anthocyanin Accumulation in Grape Berry Skin through Direct Upregulation of UDP-Glucose: Flavonoid 3-*O*-Glucosyltransferase

**DOI:** 10.3390/cells10102799

**Published:** 2021-10-19

**Authors:** Mone Yamazaki, Akari Ishida, Yutaka Suzuki, Yoshinao Aoki, Shunji Suzuki, Shinichi Enoki

**Affiliations:** 1 The Institute of Enology and Viticulture, University of Yamanashi, 1-13-1 Kitashin, Kofu 400-0005, Yamanashi, Japan; ymzkmn7@gmail.com (M.Y.); tomomisuzuki.ss@gmail.com (A.I.); yaoki@yamanashi.ac.jp (Y.A.); suzukis@yamanashi.ac.jp (S.S.); 2 Faculty of Engineering, University of Yamanashi, 4-3-11 Takeda, Kofu 400-8511, Yamanashi, Japan; yutakas@yamanashi.ac.jp

**Keywords:** anthocyanin, ethylene, grapevine, *UFGT*, sound stimulation

## Abstract

Global warming has resulted in the loss of anthocyanin accumulation in berry skin. Sound stimulation can be used as a potential method for enhancing fruit color development since many plants recognize sound vibration as an external stimulus and alter their physiological status in response to it. Sound stimulation (sine wave sound at 1000 Hz) enhanced anthocyanin accumulation in grape cultured cells and berry skins in field-grown grapevines at the early stage of ripening. The transcription of *UFGT* and *ACO2*, which encode the key enzymes in anthocyanin and ethylene biosynthesis, respectively, was upregulated in grape cultured cells exposed to sound stimulation. In contrast, the transcription of *MybA1* and *NCED1*, which encode a transcription factor for *UFGT* and a key enzyme in abscisic acid biosynthesis, respectively, was not affected by the sound stimulation. A treatment with an ethylene biosynthesis inhibitor, aminoethoxyvinyl glycine hydrochloride, revered the enhancement of anthocyanin accumulation by sound stimulation. As the promoter assay using a GUS reporter gene demonstrated that *UFGT* promoter was directly activated by the ethylene-releasing compound ethephon, which enhanced anthocyanin accumulation in grape cultured cells, we conclude that sound stimulation enhanced anthocyanin accumulation through the direct upregulation of *UFGT* by ethylene biosynthesis. Our findings suggest that sound stimulation contributes to alleviating poor coloration in berry skin as a novel and innovative practical technique in viticulture.

## 1. Introduction

Poor coloration of grape berry skin lowers the quality of grape berries. Generally, abscisic acid (ABA) controls the onset of grape berry ripening onset including anthocyanin biosynthesis in berry skin [1]. However, ABA content in grape berries is reduced by high temperatures. For example, high nighttime temperature treatment (5 °C higher than normal nighttime temperature) from before véraison to harvest decreased ABA content in berry skin, resulting in the inhibition of anthocyanin accumulation [2]. Grape berry skins exposed to a 30 °C nighttime temperature showed markedly less coloration compared with those exposed to 15 or 20 °C nighttime temperatures [3]. In 2005, a 2 °C increase in average air temperature in viticultural regions in the next 50 years was predicted using a simulation model for climate change [4]. The average temperature in Yamanashi Prefecture, a major viticultural region in Japan, has been rising by 0.042 °C every year from 1961 to date, and this trend is predicted to continue [5]. Temperature increase on a global scale will decrease areas suitable for viticulture by 25% to 73% in the Representative Concentration Pathway (RCP) 8.5 and by 19% to 62% in RCP 4.5 in major wine-producing regions by 2050 [6]. Consequently, the shift of viticultural regions northward, the establishment of vineyards at higher elevations, and the shift to wine grape cultivars tolerant to high temperatures are expected to occur in existing viticultural regions worldwide [6,7]. Global warming has shown a negative impact on berry skin coloration in current viticultural regions.

Efforts to mitigate the effects of global warming on grape berry coloration are needed in future viticulture [8]. Cluster thinning [9], trunk girdling [10], leaf removal [11], and the use of reflective mulch [12] have been adopted as cultivation techniques to increase anthocyanin content in berry skin. Misting grape bunches with water has been carried out in Chile and California to cool grape bunches [6]. The application of ABA to grape bunches after véraison has also enhanced anthocyanin accumulation in berry skin [13]. A commercial synthetic ABA (ProTone, Valent BioSciences, Libertyville, IL, USA) has been registered to enhance berry skin color without affecting other berry qualities [14]. However, there is still concern about early leaf senescence and subsequent defoliation by ABA [15,16]. In addition, these cultivation techniques demand precise application timing and intensity according to cultivar and/or field conditions as well as growers’ expertise.

The objective of this study was to investigate the effect of sound stimulation on anthocyanin accumulation in berry skin. The effects of sound stimulation on plant development and growth have long been investigated [17]. For example, sound stimulation at 1000 Hz induced the dormancy breaking of *Echinacea* seeds [18]. *Chrysanthemum* seedlings exhibited increases in root fresh weight and root length after exposure to sound stimulation at 1000 Hz [19]. Improvements in drought resistance [20] and plant defense response [21] were observed in plants exposed to sound stimulation. Preliminary experiments demonstrated that sound stimulation enhanced anthocyanin accumulation in grape cultured cells. In the present study, we investigated the effect of sound stimulation on anthocyanin accumulation in field-grown grapevines. We report herein that ethylene-mediated, but not ABA-mediated, anthocyanin synthesis is involved in anthocyanin accumulation triggered by sound stimulation.

## 2. Materials and Methods

### 2.1. Plant Materials

Anthocyanin-producing grape cultured cells, VR cells [22], were obtained from the RIKEN BioResource Center (Ibaraki, Japan). The cells were maintained at 27 °C in the dark on a modified Linsmaier and Skoog (LS) agar medium (pH 6.1) supplemented with 3% sucrose, 0.2 mg/L kinetin, and 0.05 mg/mL 2,4-dichlorophenoxyacetic acid (2,4-D). For the following experiments, VR cells were incubated on an LS agar medium without kinetin and 2,4-D in order to exclude the effects of exogenous plant hormones on anthocyanin accumulation in VR cells. 

Muscat Bailey A, a hybrid grape variety (*Vitis labruscana* (Bailey) × *V. vinifera* (Muscat Hamburg)), was cultivated in the experimental vineyard of The Institute of Enology and Viticulture at the University of Yamanashi, Japan. The grapevines were approximately 30 years old and trained to the Guyot-style training system.

### 2.2. Sound Stimulation of VR Cells

VR cells were incubated on the modified LS agar medium without plant hormones at 27 °C in the dark. The sound stimulation apparatus is shown in Figure S1. Briefly, a speaker (Onyx Studio 6, Harman International, Stamford, CT USA) was placed in an incubator (NK System Biotron LH-200, Nippon Medical & Chemical Instruments, Tokyo, Japan). Petri dishes containing VR cells were placed in the incubator at a distance of 70 cm below the speaker. VR cells were exposed to sine wave sound at 1000 Hz (sampling frequency: 44.1 kHz, quantization: 16-bit) produced using Wave Gene (http://efu.jp.net/soft/wg/wg.html, accessed on 27 July 2018) set under a laptop computer at 27 °C for 8 h in the dark. The sound pressure on the Petri dishes ranged from 90 to 95 dB. After treatment, the Petri dishes were transferred to another incubator and incubated at 27 °C for 16 h under light irradiation (11.8 Wm^−2^), followed by incubation at 27 °C for three weeks in the dark. VR cells without sound stimulation were prepared as control and incubated under the same conditions. VR cells treated with ABA were also prepared as control. Briefly, 1 mM (±)-ABA (Tokyo Chemical Industry, Tokyo, Japan) was spread all over the surface of the LS agar medium without plant hormones. VR cells were incubated on the agar medium at 27 °C for three weeks in the dark. Six independent experiments were performed.

### 2.3. Sound Stimulation of Bunches on Field-Grown Grapevine

Two field-grown grapevines were prepared for sound stimulation during the 2019 growing season. Sound stimulation was performed at véraison on 6 August 2019. A speaker (HSS-III Hypersonic Sound Audio System Model HSS-300, Videotel Digital, Chula Vista, CA, USA) was placed at a distance of approximately 70 cm from the bunches (Figure S1). The bunches were exposed to sine wave sound at 1000 Hz (sampling frequency: 44.1 kHz, quantization: 16-bit) produced using Wave Gene set under a laptop computer for 8 h (from 9 A.M. to 5 P.M.). Three bunches were randomly collected from each grapevine 0, 15, and 45 d after sound stimulation treatments.

### 2.4. Measurement of Total Anthocyanin

Anthocyanin extraction from VR cells and berry skins and its measurement were performed as described previously [23]. Briefly, ten berries (three from the top of the bunch, four from the middle of the bunch, and three from the bottom of the bunch) were collected from each bunch. Skins were peeled off from the berries using a sterilized tweezer. VR cells and skins were frozen in liquid nitrogen. One gram of the pulverized sample was macerated in 10 mL of HCl-methanol [34:1 (*v*/*v*)] at room temperature in the dark overnight. After mixing and centrifugation at 3000 rpm for 5 min, the absorbance (OD_520_) of the supernatant was measured using a spectrophotometer (UV-1800, Shimadzu, Kyoto, Japan). Total anthocyanin was calculated by using a previously published formula [24] and converted into malvidin-3-glucoside equivalent as milligrams per gram of fresh weight of VR cells or fresh skin weight, respectively.

### 2.5. Berry Characteristics

Juice was obtained by hand-pressing ten berries (three from the top of the bunch, four from the middle of the bunch, and three from the bottom of the bunch) collected from each bunch. Soluble solids (°Brix) and tartaric acid (g/100 mL) levels in the juices were measured with a refractometer (PAL-BX/ACID2, Atago, Tokyo, Japan). °Brix/acid ratio in the juice was calculated.

### 2.6. RNA Isolation

VR cells were frozen in liquid nitrogen and ground with an SK mill (SK-200, Tokken, Chiba, Japan) according to the manufacturer’s instructions. Total RNA was isolated from the pulverized samples using NucleoSpin RNA Plant (Takara, Shiga, Japan) and purified using Fruit-mate for RNA Purification (Takara), according to the manufacturer’s instructions.

### 2.7. Real-Time RT-PCR Analysis

First-strand cDNA was synthesized from total RNA using PrimeScript RT Reagent Kit with gDNA Eraser (Perfect Real Time) (Takara) according to the manufacturer’s instructions. Real-time RT-PCR was performed with TB Green Premix Ex Taq II (Tli RNaseH Plus) (Takara). PCR amplification was performed for 40 cycles at 95 °C for 5 s and at 60 °C for 45 s after an initial denaturation at 95 °C for 30 s using Thermal Cycler Dice Real Time System II (Takara). The nucleotide sequences of the primers used for real-time RT-PCR were as follows: MybA1 primers (5′-GCAAGCCTCAGGACAGAAGAA-3′ and 5′-ATCCCAGAAGCCCACATCAA-3′ from *V. vinifera MybA1*, GenBank accession no. AB111101), UDP-glucose: flavonoid 3-*O*-glucosyltransferase (UFGT) primers (5′-CTTCTTCAGCACCAGCCAATC-3′ and 5′-AGGCACACCGTCGGAGATAT-3′ from *V. vinifera UFGT*, GenBank accession no. AB047099), 9-cis-epoxycarotenoid dioxygenase 1 (NCED1) primers (5′-GAGACCCCAACTCTGGCAGG-3′ and 5′-AAGGTGCCGTGGAATCCATAG-3′ from *V. vinifera NCED1*, GenBank accession no. AY337613), 1-aminocyclopropane-1-carboxylic acid oxidase 2 (*ACO2*) primers (5′-CAAATGGACGCTGTGGAAAA-3′ and 5′-ATGGCGGAGGAAGAAGGTACT-3′ from *V. vinifera ACO2*, GenBank accession no. NM_001280942), and actin primers (5′-CAAGAGCTGGAAACTGCAAAGA-3′ and 5′-AATGAGAGATGGCTGGAAGAGG-3′ from *V. vinifera actin 1*, GenBank accession no. AF369524). Actin was used as the reference gene for data normalization. The dissociation curves were evaluated to verify the specificity of the amplification reaction. The expression levels of each gene were determined as the number of amplification cycles needed to reach a fixed threshold by the standard curve method with Thermal Cycler Dice Real Time System Single Software ver. 5.11 (Takara). Data are expressed as relative values to actin.

### 2.8. Ethephon Treatment of VR Cells

VR cells were incubated on the modified LS agar medium supplemented with 1 μM ethephon ((2-chloroethyl)phosphonic acid, Nissan Chemical, Chiba, Japan). After 3 weeks’ incubation at 27 °C in the dark, the cells were subjected to anthocyanin measurement. Two independent experiments were performed.

### 2.9. Ethylene Biosynthesis Inhibitor Treatment of VR Cells Exposed to Sound Stimulation

VR cells were incubated on the modified LS agar medium supplemented with 0.1 µM AVG (an inhibitor of ethylene biosynthesis, Fuji Film Wako, Osaka, Japan), and subjected to sound stimulation at 27 °C for 8 h in the dark as described above. The cells were incubated at 27 °C for 16 h under light irradiation (11.8 Wm^−2^), followed by incubation at 27 °C in the dark. After three weeks of incubation, the cells were subjected to anthocyanin measurement. Two independent experiments were performed.

### 2.10. Assay of UFGT Promoter in Response to Ethylene

To evaluate whether ethylene directly induced the expression of the grape *UFGT*, an assay of the *UFGT* promoter was performed. The promoter region of *UFGT*, which is 1608 nucleotides upstream of the translation start site (Figure S2) [25] containing a *Hind*III site on 5′ end and a *Bam*HI site on 3′ end, was synthesized by GenScript (Tokyo, Japan). Binary vector pCAMBIA1391Z (Cambia Labs, Brisbane, Australia), which has the β-glucuronidase (GUS) reporter gene, was digested with *Hind*III and *Bam*HI. The UFGT promoter was also digested with *Hind*III and *Bam*HI and ligated into the *Hind*III and *Bam*HI sites of pCAMBIA1391Z, resulting in a UFGTpro-GUS expression plasmid. A monolayer of onion epidermal cells (2 cm square) was bombarded with the UFGTpro-GUS expression plasmid or pCAMBIA1391Z as control using a particle gun (PDS-1000/He, Bio-Rad, Hercules, CA, USA), according to the manufacturer’s instructions. The monolayer of onion epidermal cells was incubated on the modified LS agar medium supplemented or not supplemented with 1 μM ethephon at 27 °C for 48 h in the dark. The onion epidermal cells were fixed with ice-cold 70% acetone for 15 min. After the cells were washed with washing solution (40 mM NaH_2_PO_4_, 60 mM Na_2_HPO_4_, 0.5 mM K_3_Fe(CN)_6_, 0.5 mM K_4_Fe(CN)_6_, and 0.1% Triton X-100), they were stained with GUS staining solution (40 mM NaH_2_PO_4_, 60 mM Na_2_HPO_4_, 1.25 mM K_3_Fe(CN)_6_, 1.25 mM K_4_Fe(CN)_6_, 0.1% Triton X-100, and 40 mg/mL 5-bromo-4-chloro-3-indolyl-β-D-glucuronide) at 37 °C in the dark. GUS staining in the cells was observed under a light microscope (BX51, Olympus, NY, USA).

### 2.11. Statistical Analysis

Data are presented as means ± standard errors calculated from the indicated biological replicates. Statistical analysis was performed by ANOVA variance analysis and then the Tukey multiple comparison test or the Student’s *t*-test using Excel statistics software 2012 (Social Survey Research Information, Tokyo, Japan).

## 3. Results

### 3.1. Sound Stimulation Enhances Anthocyanin Accumulation in VR Cells

**Figure 1:**
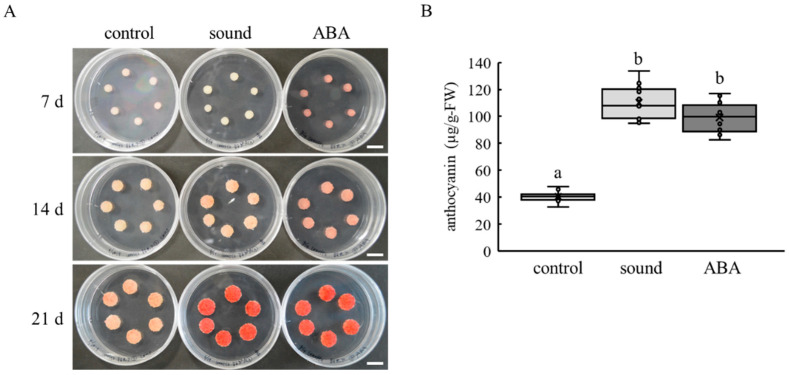
Effect of sound stimulation on anthocyanin accumulation in VR cells. (**A**) Photographs of VR cells. VR cells exposed to sine wave sound at 1000 Hz for 8 h were incubated for 7, 14, and 21 d after treatment. Scale bar: 1 cm. (**B**) Anthocyanin content in VR cells. Measurement of anthocyanin content was performed 21 d after treatment. Data indicate means ± standard errors. Crosses (×) indicate means of two independent experiments with six VR cell masses. Different letters (a, b) above the boxes indicate statistically significant differences (*p* < 0.01, Tukey). Control, no treatment. Sound, sound stimulation at 1000 Hz. ABA, 1 mM ABA treatment.

Our preliminary experiments showed that sine wave sound at 500, 2000, 4000, and 16,000 Hz had no effect on anthocyanin accumulation in VR cells (Figure S1, data not shown), whereas sine wave sound at 1000 Hz enhanced anthocyanin accumulation in VR cells (Figure 1). VR cells turned light pink 14 d after treatment and became bright red 21 d after treatment (Figure 1A). The increase in anthocyanin content in VR cells exposed to sine wave sound at 1000 Hz was comparable to that in ABA-treated VR cells 21 d after treatment (Figure 1B). The results suggest that sound stimulation at 1000 Hz enhances anthocyanin accumulation in VR cells.

### 3.2. Sound Stimulation Accelerates Anthocyanin Accumulation in Berry Skins of Field-Grown Grapevines

**Figure 2:**
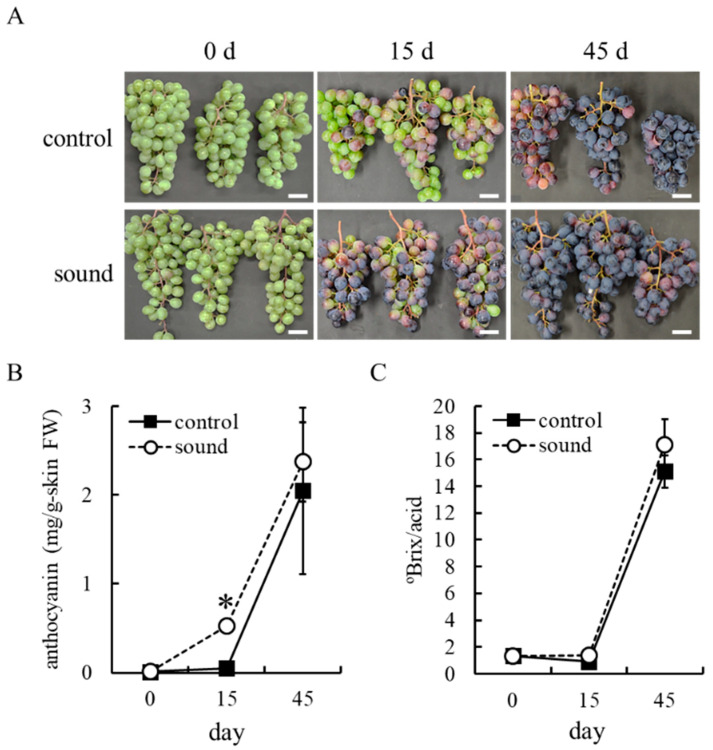
Enhanced anthocyanin accumulation in berry skins by sound stimulation. (**A**) Photographs of bunches. Scale bar: 4 cm. (**B**) Anthocyanin contents in berry skins. Data indicate means ± standard errors for three bunches. * *p* < 0.01 compared with control (Student *t*-test). (**C**) °Brix/acid ratio in juice. Data indicate means ± standard errors for juices collected from three bunches. Control: no treatment. Sound: sound stimulation at 1000 Hz.

Field-grown grapevines were exposed to sine wave sound at 1000 Hz for 8 h at véraison in the 2019 growing season (Figure S1). Enhanced berry skin coloration by sound stimulation was visible to the naked eye 15 d after treatment (Figure 2A). Berry skins exposed to sound stimulation showed an increase in anthocyanin content 15 d after treatment compared with control (Figure 2B). However, the effects of sound stimulation on anthocyanin accumulation in berry skins became negligible 45 d after treatment at harvest. The ºBrix/acid ratio during berry maturation was similar between the sound-exposed berry skins and control (Figure 2C). The results suggest that sound stimulation helps trigger anthocyanin biosynthesis in berry skin at the early stage of ripening.

### 3.3. Effect of Sound Stimulation on MybA1 and UFGT Transcription

**Figure 3:**
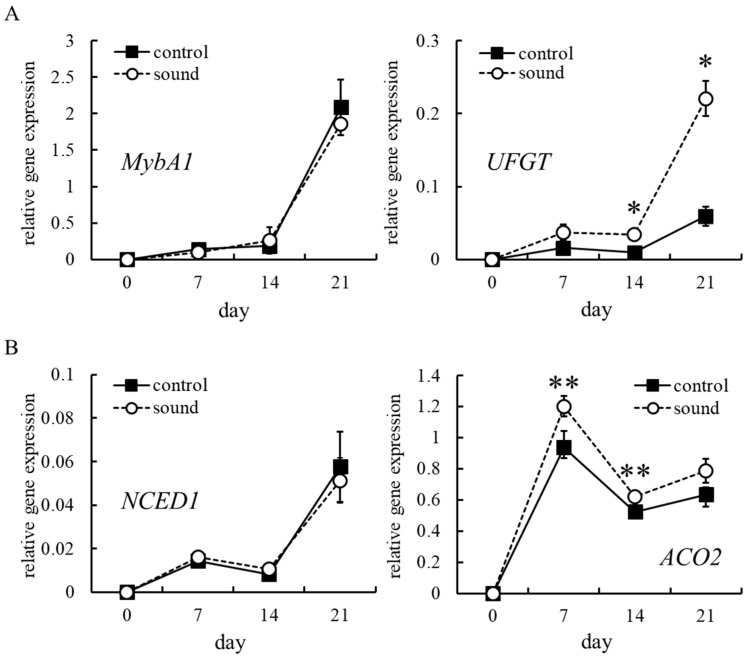
Transcription of *MybA1*, *UFGT*, *NCED1*, and *ACO2* in VR cells exposed to sound stimulation. (**A**) *MybA1* and *UFGT*. (**B**) *NCED1* and *ACO2*. VR cells were exposed to sound stimulation 7, 14, and 21 d after treatment. The transcription level of each gene was estimated by real-time RT-PCR. Data were calculated as gene expression relative to *actin* expression. Data indicate means ± standard errors of two independent experiments with three VR cell masses. * *p* < 0.01, ** *p* < 0.05 compared with control (Student *t*-test). Control: no treatment. Sound: sound stimulation at 1000 Hz.

UFGT, which catalyzes anthocyanidin glycosylation, is the most important enzyme for anthocyanin accumulation in berry skin [26]. *UFGT* is transcribed by transcription factor MybA1, which determines berry skin color [27]. To determine whether sound stimulation upregulates *MybA1* and *UFGT* transcription for enhanced anthocyanin accumulation, the transcription level of each gene in VR cells exposed to sound stimulation was measured. The upregulation of *MybA1* transcription was not observed relative to control (Figure 3A). In contrast, sound stimulation upregulated *UFGT* expression by approximately 350% 14 d and 21 d after treatment compared with control (Figure 3A). These results suggest that sound stimulation induces *UFGT* transcription without upregulating *MybA1* transcription.

### 3.4. Effect of Sound Stimulation on ABA and Ethylene Biosynthesis

Exogenous stress treatment activates ABA biosynthesis and then ABA upregulates *MybA1* transcription [28]. As ABA biosynthesis is activated by the upregulation of *NCED* transcription [29], we examined whether sound stimulation upregulates *NCED1* transcription, and found that sound stimulation did not increase *NCED1* transcript levels in VR cells (Figure 3B). 

The upregulation of UFGT transcription by ethylene is independent of *MybA1* transcription [30]. Ethylene synthesis from 1-aminocyclopropane-1-carboxylate (ACC) is catalyzed by ACC oxidase (ACO) [31]. VR cells exposed to sound stimulation 7 and 14 d after treatment showed upregulated *ACO2* transcription compared with control (Figure 3B).

**Figure 4:**
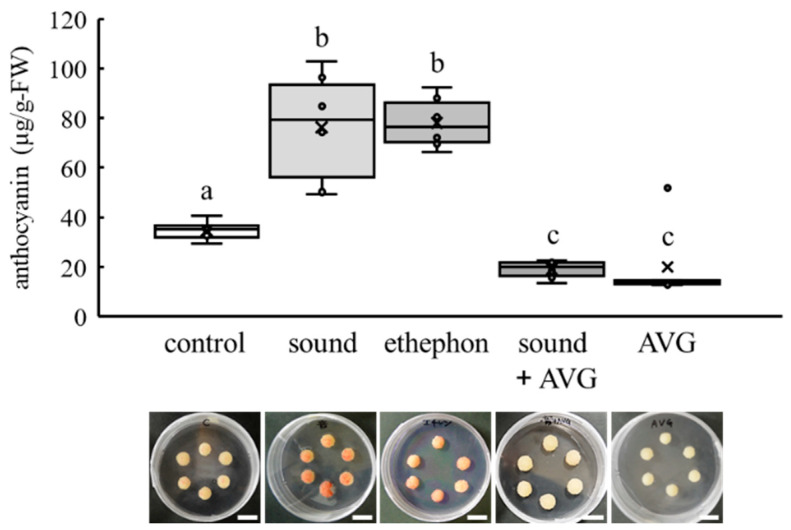
Effect of ethephon and AVG on anthocyanin accumulation in VR cells exposed to sound stimulation. VR cells were treated with ethephon, an ethylene-releasing compound. Another group of VR cells exposed to sound stimulation were treated with AVG, an inhibitor of ethylene biosynthesis. Anthocyanin content was measured 21 d after treatment. Data indicate means ± standard errors. Crosses (×) indicate means of two independent experiments with six VR cell masses. Different letters (a,b,c) above the boxes indicate statistically significant differences (*p* < 0.01, >Tukey). Representative VR cells are shown below the graph. Scale bar: 1 cm. Control: no treatment. Sound: sound stimulation at 1000 Hz. Ethephon: 1 μM ethephon treatment. Sound+AVG: sound stimulation at 1000 Hz and 1 µM AVG treatment. AVG: 0.1 µM AVG treatment.

Ethephon, an ethylene-releasing compound, enhanced anthocyanin accumulation in VR cells (Figure 4). In contrast, aminoethoxyvinyl glycine hydrochloride (AVG, an inhibitor of ethylene biosynthesis) inhibited the enhancement of anthocyanin accumulation by sound stimulation (Figure 4).

Taken together, these results suggest that the upregulation of *UFGT* transcription, followed by the acceleration of anthocyanin biosynthesis by sound stimulation may occur through the activation of ethylene biosynthesis, but not ABA biosynthesis.

### 3.5. Activation of UFGT Promoter by Ethephon

**Figure 5:**
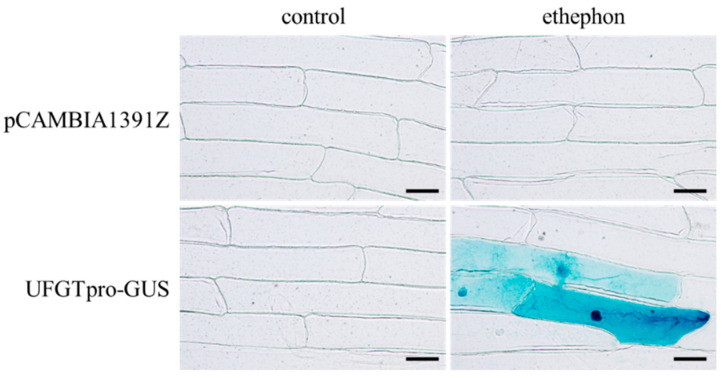
Activation of *UFGT* promoter by ethephon. After ethephon treatment, transformed onion epidermal cells with UFGTpro-GUS expression plasmid or pCAMBIA1391Z were subjected to GUS staining assay as described in Materials and Methods. Blue color shows 5,5′-dibromo-4,4′-dichloro-indigo produced by GUS. The results represent reproducible data from at least three independent experiments. Scale bar: 100 μm. Control: no treatment. Ethephon: 1 μM ethephon treatment.

To determine whether ethylene directly activates *UFGT* transcription, the promoter assay was performed. GUS reporter gene regulated by UFGT 1,608 bp promoter upstream of the translation start site (Figure S2) was transformed into onion epidermal cells using the UFGTpro-GUS expression plasmid. After ethephon treatment, onion epidermal cells transformed with the UFGTpro-GUS expression plasmid showed blue color produced by GUS (Figure 5). The upregulation of GUS reporter gene was not observed in onion epidermal cells transformed with pCAMBIA1391Z. Predicted ethylene-responsive element ATTTCAAA [32] was detected in the *UFGT* promoter (Figure S2).

Taken together, this result emphasizes that sound stimulation enhances anthocyanin accumulation through the direct upregulation of *UFGT* by ethylene biosynthesis.

## 4. Discussion

**Figure 6:**
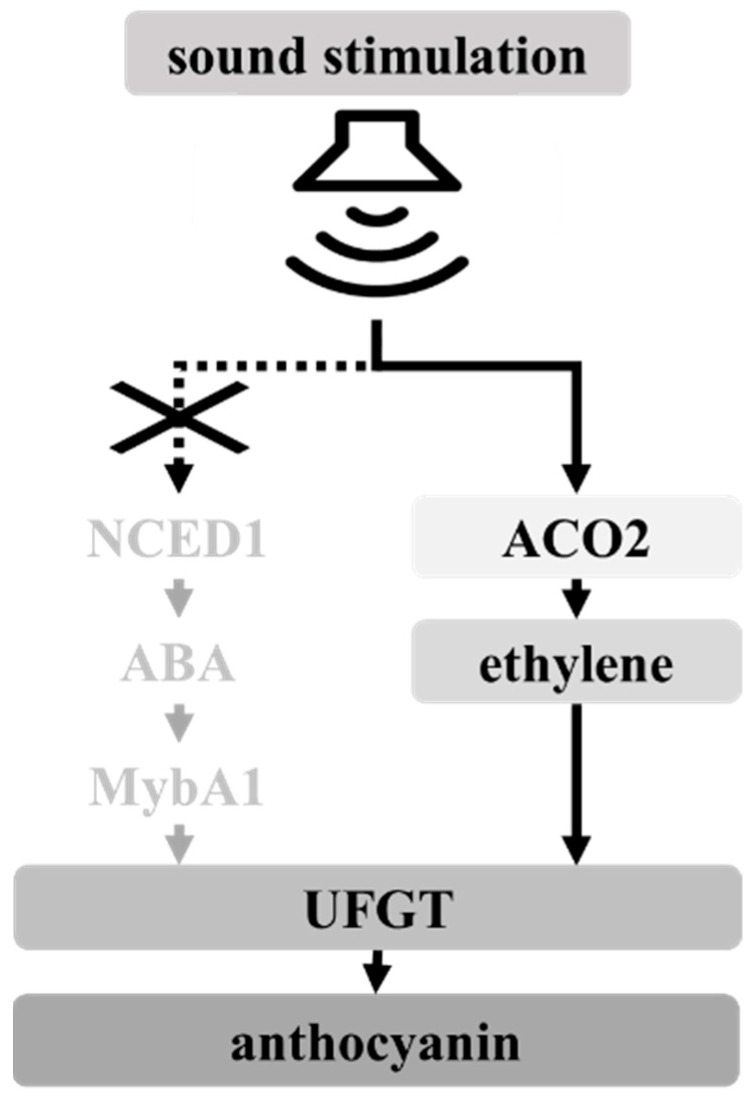
Predicted pathway for enhancement of anthocyanin accumulation by sound stimulation.

A predicted pathway for the enhancement of anthocyanin accumulation by sound stimulation is shown in Figure 6. ACO is a key enzyme for ethylene biosynthesis [31]. Generally, ethylene biosynthesis is rapidly activated by stress stimulation [33]. However, sound stimulation upregulated *ACO2* transcription in VR cells 7 and 14 d after treatment (Figure 3B). Ethylene content in grape berries (at véraison) treated with the ethylene-releasing compound ethephon was increased within 24 h after treatment [34], whereas the long-term expression of *UFGT* and other genes related to anthocyanin biosynthesis in grape berries persisted more than 20 d after treatment. As the upregulation of *UFGT* transcription and the enhanced anthocyanin accumulation were observed in VR cells 14 d and 21 d after sound stimulation (Figure 1 and Figure 3, respectively), sound stimulation might work as a slow-acting elicitor of anthocyanin accumulation through ethylene biosynthesis.

Ethylene induced the transcription of genes, including *UFGT*, related to anthocyanin biosynthesis in grape berries [34,35]. As grape berries are classified as a non-climacteric fruit [36], grape berry ripening is said to have long occurred independently of ethylene [37]. There is no question that ethylene is critical for some berry-ripening processes including anthocyanin accumulation in berry skin [38]. For anthocyanin biosynthesis, the upregulation of *UFGT* transcription by ethylene is independent of *MybA1* transcription induced by ABA [30]. In the present study, we found that (1) sound stimulation did not upregulate *NCED1* and *MybA1* transcription (Figure 3); (2) ethephon induced anthocyanin accumulation (Figure 4); and (3) *UFGT* promoter, having a predicted ethylene-responsive element (Figure S2), was activated by ethephon (Figure 5). Therefore, we predicted that sound stimulation enhanced anthocyanin accumulation through the direct upregulation of *UFGT* by ethylene, but not through the upregulation of *MybA1* by ABA, as shown in Figure 6. Although we could not determine the quantities of ethylene in VR cells and berry skin exposed to sound stimulation due to technical difficulties, our prediction was supported by the finding that the enhancement of anthocyanin accumulation in VR cells exposed to sound stimulation was suppressed by inhibiting ethylene biosynthesis (Figure 4).

Sound stimulation produces sound vibrations. The primary target of sound vibration may be the plasma membrane [39]. Sound vibration targets the plasma membrane and evokes a signal transduction cascade through the cytoskeleton or ion channels [40]. Ca^2+^ may act as a secondary messenger of sound vibration, passing signals through phosphorylation/de-phosphorylation to other signaling proteins or transcription factors [21]. Sound vibration also modulates gene expression through chromatin remodeling [41]. Finally, gene expression is activated by sound vibration, resulting in physiological and developmental alteration in plants [42,43]. In grapevine cells, *ACO2* was one of the target genes induced by sound vibration. Ethylene produced by *ACO2* directly activated *UFGT* transcription followed by anthocyanin accumulation in grapevine cells. However, we were unable to demonstrate any results related to other physiological changes in grapevines. In the field trial, macroscopically observable negative effects of sound stimulation on the growth of shoot, leaves, and bunches were not apparent (data not shown). To comprehensively determine the positive and negative effects of sound stimulation on grapevine physiology, future studies employing RNA sequencing or microarray analyses should be conducted to reveal transcriptome alterations in grapevine in response to sound stimulation. In addition, to further understand molecular signaling from sound stimulation to anthocyanin accumulation, quantitative and histochemical analyses of Ca^2+^ in grapevine cells exposed to sound stimulation are required.

What about sound stimulation as a new technique for improving grape berry coloration? Recently, viticulturists have expressed concern regarding the poor coloration of grape berries due to global warming [44]. Many researchers have proposed new techniques for improving anthocyanin accumulation in berry skin using chemical [13,28,45], physical [46], and biological [47,48] approaches. Our sound stimulation is categorized as a physical approach for improving grape berry coloration. Compared with the chemical approach, which is easily executed by spraying, the physical approach by sound stimulation requires the development of a universal sound stimulation apparatus to ensure that the approach works effectively. On the other hand, several reports claimed that sound stimulation had negative effects on plant development. For example, tomato fruit exposed to sound stimulation at 1000 Hz showed delayed ripening due to the downregulation of ethylene biosynthesis [49]. Further field trials are necessary to clarify the optimum conditions for the sound stimulation of each cultivar and each cultivation environment in vineyards.

## 5. Conclusions

Our findings highlight the application of sound stimulation as an eco-friendly viticultural technique for improving grape berry coloration. We demonstrated that sound stimulation enhanced anthocyanin biosynthesis by promoting ethylene biosynthesis, and that exposing bunches to sound stimulation at véraison enhanced anthocyanin accumulation in berry skin at the early stage of ripening. Future studies of postharvest sound stimulation of poorly colored bunches could reveal the potential commercial application of sound stimulation in viticulture.

## Supplementary Materials

The following are available online at https://www.mdpi.com/article/10.3390/cells10102799/s1, Figure S1: Experimental design of sound stimulation of grape cultured cells and field-grown grapevines. Figure S2: Nucleotide sequence of *UFGT* gene for promoter assay.
