# Supplementary material for: Ethylene Induced by Sound Stimulation Enhances Anthocyanin Accumulation in Grape Berry Skin through Direct Upregulation of UDP-Glucose: Flavonoid 3-O-Glucosyltransferase
Source: Cells. 2021 Oct 19;10(10):2799. doi: 10.3390/cells10102799 (PMC8534375; doi:10.3390/cells10102799)
Supplement: Supplementary file 1 [file cells-10-02799-s001.zip › cells-1428515-supplementary.pdf]

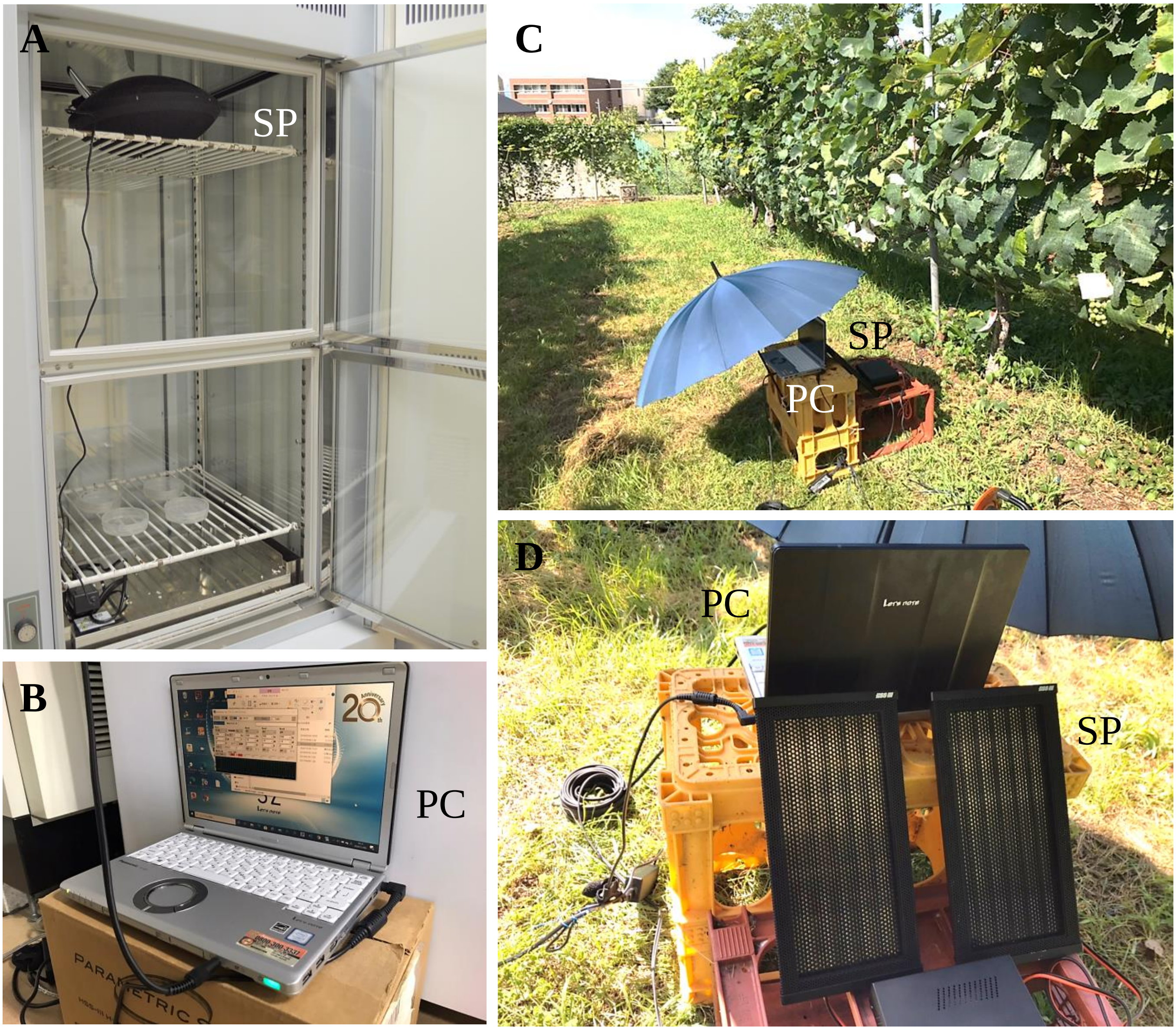

Figure S1. Experimental design of sound stimulation of grape cultured cells and field-grown grapevines. (A) Sound stimulation of VR cells. A speaker (SP) was placed in an incubator. Petri dishes containing VR cells were placed 70 cm below the speaker. (B) Sine wave sound at 1,000 Hz was produced using Wave Gene (http://efu.jp.net/soft/wg/wg.html) under a laptop computer (PC). (C) Sound stimulation of bunches on field-grown grapevine. Sound stimulation was performed at véraison (August 6, 2019). A speaker (SP) was placed approximately 70 cm away from bunches. (D) Sine wave sound at 1,000 Hz was produced using Wave Gene set under a laptop computer (PC).

−1608	CTCATCTTGCACATCCAATGATTGGTATGAATTCAACAGTACCTAATA	−1561
−1560	GCACAATACTATAGAATTCTCCCTAAATTTATGAAAAAATTACATTTTTGTAAGATCTAT	−1501
−1500	CTAACCTCTGATTCAGCACACTCTAGAGCAACATTGAGACAACTGCAGGCTGCAGCTTTT	−1441
−1440	TCAACAATCTCAATTTTGCCCCAAAAATTTCCAAACATGGAAACCAGAAAATATTGTGAC	−1381
−1380	TTGTTAAGGGCTTTTTCGTCATTTCATCTTGGTTTTATTTGACATCATTTTTTTTAAATT	−1321
−1320	GTCTTTAAGAACAAAATTTATCTAGGAACATAAATATGAAAAACGATATTTTGGTCTTAT	−1261
−1260	TCCACATGAGGGTGTTGTATAATTATATGCAATAAAAAAATTGTTGAAATATTTTTCATA	−1201
−1200	TTTCAATTGTTATCTAAAATACTTTACAAAAAAACGATTGAGAGCATATTAGACTTGGTC	−1141
−1140	TTAAAAATAACTTGTTTTTAGAACACATTCTCAAAAAAAATATTTTCTGTAAAAAATTTA	−1081
−1080	GGGTCTGTTTGATAACTATTTTCTAAAACAGTTATAAAAAATAGTTTTTTAAAACTTTTC	−1021
−1020	TCTAATGTTTTGTAAAACAAAAATTTGTTTATAAATCTAAAATATTTTAACATATTTTTA	−961
−960	ATATTTTTTTTAGTATATTTTAAAAATAAGTTTTATATCTAGTATTTTATTTTTAATCAT	−901
−900	TCTACATATTTGTATAATTATTTTTCAAAATAACCATAAAAAAACAGTTAAAAGAAATTT	−841
−840	TCTGAAAATACCGTATTTTTTTTATCTAAATAGTTTTTTATTGTTAAATATATTATCCTA	−781
−780	GTTTTTTATTTTAAAGAATAGAAAACCATTCTCGGAAAATAAATACCAAATAGACTCTTA	−721
−720	CTTTTTAAATTAAAATACAGTTTTTGGGTCTTTTCAAAAAAAGTTTTCAAACAAGTCCTT	−661
−660	TAGTTTCTTTAATTATTTGCTTTTTCAAAATCAATTCTTTAAAAATATTTTTTGAAAATA	−601
−600	ATTCTTTAAAATATTTTTCAATTGTTTTTTGGTAACAAAATTCTATCTAATAACCAAAAT	−541
−540	ATAAAAAATATTTTTTTTAGTTCTCTTCATAAAAGTAATATATATCATGTGGAATACAAA	−481
−480	AACTTTTAAAACATTCTCTACCTTTTCAATATTACTAAAAAAAACAATTTTAAATTATTC	−421
−420	TTAAAACAATGGGGTTTTAATCAAATTAATTTTGAAAACATTAATTTATTTCAAAAAATT	−361
−360	TATTGAATCATATTTTTCAAATTAGAAAACAATTTTATGTTGTTTAGAATAGAAAATTAT	−301
−300	TTTTGAAGTAAAATTAACAAATATGCTCTTGCTACTACTCGCCAAGTATATCCCACCAAT	−241
−240	GGCAAAGTAAAAGCTCACACAGAGCTTTCACTTGCCCCTGGTTTTGTTTTTTTTTTCCCA	−181
−180	TTTTTTCTTTCTTTGGCCGCCATGCAGAATGGTGGTTTGGTTTTGGGTTGGTTTGTTAGG	−121
−120	AGGGTGGGAATTGGGATGACAACCCCCATGCAGTTGCCACTCTCACAACCCCCATGCAGT	−61
−60	TGCTCTCATTTATAATCTTCAACAGCCAAAACCCAAATTGTAACATCCCCCATTCCAACC	−1

Figure S2. Nucleotide sequence of *UFGT* gene for promoter assay. A 1,608 bp promoter upstream of the translation start site (*V. vinifera* UDP-glucose: flavonoid 3-*O*-glucosyltransferase gene, promoter region, GenBank accession no. AY955269. Kobayashi et al., 2001) was used. Black shaded region is a highly conserved region among *Vitis* species. Boxed 8-bp sequence (ATTTCAAA) is the predicted ethylene-responsive element (Itzhaki et al., 1994).
